# Family Relationships in Selective Mutism—A Comparison Group Study of Children and Adolescents

**DOI:** 10.3390/children9111634

**Published:** 2022-10-27

**Authors:** Siebke Melfsen, Thomas Jans, Marcel Romanos, Susanne Walitza

**Affiliations:** 1 Psychiatric University Hospital Zurich, 8032 Zurich, Switzerland; 2 Department of Child and Adolescent, Center of Mental Health, University Hospital of Wuerzburg, 97080 Wurzburg, Germany

**Keywords:** selective mutism, family relations, parents, schoolchildren

## Abstract

Selective mutism (SM) mostly develops early in childhood and this has led to interest into whether there could be differences in relationships in families with SM compared to a control group without SM. Currently, there are merely few empirical studies examining family relationships in SM. A sample of 28 children and adolescents with SM was compared to 33 controls without SM. The groups were investigated using self-report questionnaires (Selective Mutism Questionnaire, Child-Parent Relationship Test—Child Version) for the assessment of SM and family relationships. Children with SM did not report a significantly different relationship to their mothers compared with the control group without SM. However, the scores in respect to the relationship to their fathers were significantly lower in cohesion, identification and autonomy compared with children without SM. Relationships in families with SM should be considered more in therapy.

## 1. Introduction

Selective mutism (SM) is categorized as an anxiety disorder. It is characterized by a persistent failure to speak in specific situations, whereas in other situations the sufferers speak quite normally. To meet diagnostic criteria the symptoms need to occur for more than one month and should not be limited to the first month of school [1]. Not only the ability to speak is impaired in SM but other areas of behaviour are also impacted. Typical symptoms of SM are reduced gesture and facial expressions, cramped-looking postures, limited movements, gaze aversion and changes to the sound of their own voice as well as shut-down or freezing in specific settings [2]. The prevalence rate of SM is reported to range from 0.03% to 2% [3,4,5,6]. SM typically begins very early, first becoming apparent in unfamiliar situations such as the beginning of nursery or school.

Parents of children with SM face completely different challenges than parents of children without SM. Seeing their child frustrated by being unable to speak in certain situations may spread feelings of helplessness as well as lack of understanding, which in turn may change and negatively impact family relationships [7]. 

Unfortunately, research regarding psychological profiles in family members, familial relations and environment in SM is sparse and there are only a handful of existing studies. A family study conducted by Capozzi et al. [8] evaluated the psychological profiles of preschool children with SM as well as their parental psychological profiles, and compared them to the profiles of children with generalized anxiety disorder (GAD) and their parents’ profiles. Children with SM scored higher on the “CBCL-1½-5 withdrawn scale” and lower on the “attention problems”, “aggressive behaviour”, and “externalizing problem” scales than children with GAD. Mothers of children with SM scored higher on “SCL-90-R obsessive-compulsive behaviour” while fathers of children with SM scored higher on “phobic anxiety” than parents of children with generalized anxiety disorder. Therefore, apart from genetic components predisposing families of children with SM [9], several other shared familial factors may be implicated such as anxious behaviour modelling by parents. It is not seldom that one family member of children with SM is the “silent type” and is not verbally expressive [10,11,12]. Two other studies [13,14] found no association between SM and maternal psychopathology but paternal psychopathology. In a new study by Koskela et al. [15], potential risk factors for SM were examined. The results show that parental psychology, the parents’ age and the marital status of the mothers increased the odds of SM. 

The familial relations were often described as conflict-laden and isolated. In such studies, mothers have been characterized as “over”: they tended to be over-protective, over-controlling and over-involved, whereas fathers were mostly described as detached [16,17,18,19,20,21,22,23,24,25]. Edison et al. [18] examined parent-child interactions among SM, anxious and non-anxious children in different contexts. In all contexts, parents of children with SM were more controlling than parents of the other groups. The authors proposed that parents intervene for their children in case of failing to meet performance demands. In a study done by Buzzella et al. [26], parents of children with SM also reported more often to monitor their children’s activities than families of children without significant anxiety or oppositional behaviour, and no other differences in parenting behaviours were reported.

In some studies, the mother and child with SM were in close relationship, while the relationship with the father was described as peripheral and passive [27,28]. However, the study results are divergent and could be explained by different research methods. Studies using self-report inventories have failed to find differences in parenting styles between families of children with SM and controls [29,30]. They demonstrated that the child’s family shows an adequate competence [14,27]. However, in some studies using self-report inventories for children with SM, less warmth and acceptance from their parents were reported compared to controls [29].

An important aspect concerns the external environment. In some studies, the family members of children with SM tended to perceive it as threatening. Social isolation of families was also reported in SM [10,11,12,13]. 

Furthermore, parents of children with SM reported more stressful life events [8]. Accordingly, in a study by Elizur and Perednik [19] higher rates of marital conflicts could be observed. In several other studies, family stressors such as divorce or death were reported [10,11,12,13,15].

Patterns of relationships between family members and the children with SM as well as the family environment presumably play an important role in the aetiology and maintenance of the disorder. However, only little is known about family relations and environment in SM. The present study aims to investigate the child’s perspective of family relationships, as this perspective may have more impact on child development than the parental one. We aim to investigate how children and adolescents with SM rate parent-child relationship quality in comparison to control participants without SM. Because of the inconsistent study results concerning mother-child relationship, we do not issue any directed hypotheses, but rather assume differences to the control group without SM. For the father-child relationship we assume a peripheral relationship.

## 2. Materials and Methods

### 2.1. Procedure

The present clinical observation study compares a sample of children and adolescents with SM (MG) to a group of children and adolescents without SM (CG) regarding various aspects like emotion regulation [31], sensory-processing sensitivity and dissociation [32] as well as family relationships. Participants were recruited at the Mutism Special Outpatient Clinic of the University of Dortmund, the Department of Child and Adolescent Psychiatry, Psychosomatics and Psychotherapy of the University of Wuerzburg and Department of Child and Adolescent Psychiatry and Psychotherapy of the University Hospital Zurich as well as in cooperation with two non-profit advocacy groups (www.mutismus.de, accessed on 18 October 2022 and stille-staerken.de, accessed on 18 October 2022), and psychotherapeutic outpatient practices. The control group was recruited through leaflets at sports clubs and youth facilities. A small compensation for participation were offered to all controls [31,32].

Questionnaires were delivered to each participant either in person or through postal mail and were completed at home. The average duration to complete the questionnaire was one hour each for the child and the mother. After that, the questionnaires were sent back to the investigators [31,32].

### 2.2. Participants

Participants aged 7 to 18 years were included if they met DSM-5 diagnostic criteria for SM according to the information in the medical history sheet in addition to a diagnosis previously made by a psychiatrist or psychologist and a diagnostic cut-off by the “Selective Mutism Questionnaire (SMQ)”. In addition to this, a sufficient command of the German language was also required. Participants were excluded if they reported pervasive developmental disorders or communication disorders that could better account for the child’s symptoms. All participants lived in Germany or Switzerland [31,32].

### 2.3. Measures

**Medical history sheet**: First, basic demographic characteristics and information regarding the child were collected: age, sibling rank, gender, developmental delay or disorder in the acquisition of language, the child’s course of SM, co-morbidities.

**Family characteristics**: In the medical history sheet, mothers were asked about socio-demographic variables like nationality, family structure (marriage/cohabitation; separation/divorce; single) and their valuation of family relationship structure—mother’s perspective (as good, mediocre or bad).

**Selective mutism**: The “Selective Mutism Questionnaire (SMQ)” [33,34,35] (is the German version of a parental report measure assessing symptoms of SM, severity, range and functional impairment. The questionnaire is a 17-item parent-rating measure. The frequency of the speaking behaviour is rated, based on a 4-point scale from 0 (never) to 3 (always) at school (6 items), at home/in family (6 items) and in public settings outside school (5 items) with a total score of 51. For the German version, a forward-backward translation has been carried out. Its internal consistency is satisfactory in the range of α = 0.83–0.96; N = 179. Its total scale reliability is α = 0.95. A group of 96 children and adolescents with SM and 80 children and adolescents without SM were compared. The results clearly showed that the SMQ total value differed significantly between the group with SM (M = 19.08; SD = 7.49) and the group without SM (M = 42.39; SD = 8.71; t(0.95; 169) = 18.88; *p* < 0.001; d = 2.90). In addition, the structure of the English version with three factors has been confirmed for the German version (Melfsen & Walitza, in preparation) [31,32].

**Family relationship structure—child’s perspective**: The “Child-Parent Relationship Test-Child Version (ChiP-C)” [34] is a self-report questionnaire that is used to record the quality of the child-parent relationship according to the child’s appraisal. The participants are asked to rate 36 items on a five-point Likert scale (0 = never, 4 = always) representing three resource scales (“Cohesion”, “Identification”, “Autonomy”), five risk scales (“Conflict”, “Punishment”, “Rejection and Indifference”, “Emotional Burden”, “Overprotection”), and one additional scale (“Help for the Parents”). 

The “Cohesion” scale measures parental emotional warmth and bonding, intimacy, mutual support, care and reliability (“I’ve had the feeling my mother/father really loves me”) with four items. The “Identification” scale addresses an adolescent’s sense of being like a parent or the acceptance of the parents as role models (“I want to become just like my…”) with four items. The “Autonomy” scale assesses the granting of appropriate autonomy and mutual influence between children and parents (“My… has allowed me to decide for myself”) with four items. The “Conflict” scale assesses how often the child has experienced conflicts with his or her parents (“My… has argued with me”) with four items. The “Punishment” scale assesses physical violence or inappropriately severe punishment (“My mother/father beats me”) with three items. The “Rejection and Indifference” scale assesses the child’s feelings of being openly or covertly rejected or neglected by a parent (“My mother/father told me I am useless”) with four items. The “Emotional Burden” scale addresses personal fears, sorrows and problems shared by a parent with a child and perceived by the child to be a burden (“I had to console my mother/father”) with four items. The “Overprotection” scale indicates undue and exaggerated parental fears and worries about the child and overprotective parenting behaviour (“My mother/father has been very anxious about me”) with four items. The “Help for the Parents” scale indicates how often a child feels that his or her parents need help for everyday responsibilities (“I have to relieve my mother/father of tasks”) with four items [36].

All items are rated for maternal as well as for paternal behaviour. The rating is evaluated via the mean score. The overall quality of the parent-child relationship is assessed using an index value. The discrepancy between the representations of the mother and the father is recorded with a second index value. The coefficients of internal consistency lie between 0.70 and 0.80 for the subscales. For the retest reliability, 47 participants were examined again after an average of seven weeks and demonstrated retest coefficients between 0.65 and 0.91. The ChiP-C is known to have good validity and construct validity [36].

### 2.4. Statistical analyses

For data analysis we used the statistical programming package R [37]. In order to calculate the sample size for *t*-test/variance analyses procedures, we specified a significance level of *p* < 0.05, a power of 0.08 and an effect size of 0.5. A sample size of 27 children/adolescents per group was calculated. Furthermore, we did a screening for correctness of data entry. Before statistical analyses were done, checks for normal distribution and missing values checks were carried out. The minimum completion rate for questionnaires only partially filled in was set to 90%. In order to find out differences between groups we used *t*-tests as well as appropriate non-parametric tests.

## 3. Results

### Participants

The sample MG consisted of 28 children and adolescents with SM. They were aged 7 to 18 years with a mean age of 12.66 years (SD = 3.98). Eighteen participants were females. The majority’s mother tongue was German (89.29%). The SM group showed a high rate of co-morbid mental disorders and developmental abnormalities (Table 1).

The control group CG without SM comprised 33 pupils aged 7 to 18 years with a mean age of 12.45 years (SD = 3.18). Twenty-one of the participants were female. The majority’s mother tongue was German (97%) (Table 1).

**Selective mutism**: In the SMQ [33] children and adolescents with SM (n = 28) showed a low score, indicating a high level of SM symptoms (M = 20.91, SD = 8.08). Accordingly, in the control group (n = 33), the score was significantly higher with a mean of M = 42.76 (SD = 11.28) indicating a low level of SM-symptoms. The median test showed a significant group difference (X^2^(1) = 29.70, *p* = 0.0001) and a strong effect was indicated by the effect size of d = 2.19 [31].

**Medical history sheet**: 24 mothers of children with SM reported problems in early development like physical illnesses, mild developmental delays or emotional problems. There were only six mothers of the control group reporting comparable problems. 

**Family characteristics**: In the SM group, there was no higher number of divorced parents (28.6%) in comparison to the control group (27.3%). 

**Family relationship structure—mother’s perspective**: Within the SM group, a high percentage of mothers rated the relationship between father and the child with SM as bad (28.6%, n = 8) or as mediocre (25%, n = 7). In the control group, only 18.2% (n = 6) of the mothers rated the relationship between father and participating child as bad and 6.1% (n = 2) rated it as mediocre (Table 2).

**Family relationship structure—child’s perspective:** There was a significant discrepancy in the perceived relationship with the mother and the perceived relationship with the father between children with SM and the control group in the ChiP-C [38]. This discrepancy was assessed in terms of cohesion (MG: M = 0.97, SD = 0.83; CG: 0.45, SD = 0.54; F (1, 34) = 6.43, *p* < 0.05, d = 0.76), the number of conflicts (MG: M = 0.85, SD = 0.76; CG: M = 0.49, SD = 0.38; F (1, 28) = 4.22, *p* < 0.05, d = 0.63) and overprotection (MG: M = 0.97, SD = 0.91; CG: M = 0.52, SD = 0.44; F (1, 28) = 4.55, *p* < 0.05, d = 0.65).

Children and adolescents with SM did not report significantly higher or lower scores in respect to the relationship with their mothers compared to the control group on the ChiP-C (Table 3). However, in children with SM, significantly lower cohesion scores were observed with the fathers (MG: M = 2.05, SD = 1.07; CG: M = 2.78, SD = 1.01; W = 408, *p* < 0.05, d = 0.69), which comprised less emotional closeness, less positive physical contact, less instrumental and social support. Furthermore, significantly lower identification scores were observed with the fathers (MG: M = 1.95, SD = 0.91; CG: M = 2.72, CG = 0.84; X^2^ (1) = 6.23, *p* < 0.01, d = 0.88), which includes the role model function and the perceived and desired similarity to the father. In addition, a significantly lower degree of autonomy was reported, which related to the extent of own decision-making, the father’s confidence in his child’s decisions and the opportunities to assert important interests (MG: M = 2.13, SD = 0.76; CG: M = 2.63, SD = 0.74; X^2^ (1) = 11.01, *p* = 0.001, d = 0.67). Overall, the quality of the relationship with the father was reported as less close than in controls (MG: M = 2.81, SD = 3.08; CG: M = 4.94, SD = 3.28, X^2^ (1) = 4.71, *p* < 0.05, d = 0.67). However, no significant differences on the other ChiP-C-scores were reported between children with SM and the control group concerning the relationship to their fathers.

## 4. Discussion

The present study investigates whether children and adolescents with SM differ in their relationships with their parents from a control group without SM. The previous literature has generally suggested that mothers of children with SM are believed to be over-protective, over-controlling and over-involved, whereas children’s fathers are described as detached (e.g. [18,19,23]. In one study, the parents were asked to assess their own relationship to their children. Only higher levels of monitoring of the children’s activities were reported [26]. However, when assessing the children with SM themselves, less warmth and acceptance from the parents were reported [29,30].

The present study collected data directly from children and adolescents with SM who completed self-report questionnaires. There was a significant discrepancy in the perceived relationship with mothers and fathers in the group of children and adolescents with SM compared to the control group. The relationships to the mothers of children with SM were not perceived as significantly different to the control group. However, the scores in respect to the relationship to their fathers were significantly lower in cohesion, identification and autonomy. The children and adolescents with SM reported experiencing less emotional closeness, less positive physical contact and less support by their fathers. The father’s model function was reported as less pronounced. Less confidence in the children’s decisions was perceived. The results are in line with a previous case study that demonstrated that the relationship between a child with SM and their mother was close, while a peripheral and passive relationship was described between the child and their father [27].

These results are comparable with findings from the present study of mothers predominantly rating their relationship with their child with SM as good, while the relationships with the fathers were more often rated as mediocre or bad. 

This finding may be related to the stress and burden caused by the disorder of SM. The stress may lead to strain within familial relationships. This interpretation is in line with research that focuses on the bidirectional nature of parent-child interactions rather than focusing on specific parental behaviours [39]. The study of Nowakowski et al. [39] investigated joint attention behaviours. The children with SM withdraw from interactions with their parents. This could not be observed in children with other anxiety disorders. The study showed that the children with SM have been less responsive to their parents’ communicative acts, thus leading to a breakdown in the parent-child communication and thus missing out on opportunities to learn other coping and problem-solving skills.

For future studies, it will be important to assess the parents’ perspective in parallel to that of the children in more detail, as the assumption of a causal association is still unexplored. It will be important to inquire about the father’s perspective on the family relations. It will be also important to ask the mothers how they rate their own relationship with their husbands or partners. 

If the results of the present study were to be replicated, the reasons for the fathers’ role should be investigated. Is it, for example, the result of an above-average stressful everyday situation, which especially mothers and their children with SM experience? Does the need for the child’s protection lead to an emotional distance to the father?

Generally, parents want to support good and healthy development by creating an environment in which the necessary learning processes can take place [40]. However, this task becomes more difficult if it is unclear for them which environment may be helpful for the child with SM and its learning processes. Our results demonstrate the need for increased availability of support for the parents of children with SM, such as information regarding how to manage SM. Such support sources are likely to have a positive impact on the relationships between children with SM and their families.

The risk of disruptions to typical developmental trajectories is greater if there is a lack of emotional attachment and autonomy to one or both parents, as this may impact the families’ ability to endure burdens and to cope with crises [41]. However, only long-term studies can clarify whether a detached relationship to one’s parents may be an aetiological factor of SM [16].

Current research shows that parental overprotection and control may be associated with anxiety disorders [42]. Thus, the family factors of children with SM and of children with anxiety disorders seem to differ.

It would also be beneficial for future studies to examine the long-term course of these relationships in order to be able to assess their development. Furthermore, assessing relationships should not be limited to parents, but sibling relationships should also be taken into account. It is also important to take a holistic view of families instead of a deficit one by examining family satisfaction and family quality of life. 

The present study has some limitations. Because of the low prevalence of SM, the sample recruited for the study is small. Consequently, for verification the results of our study should be replicated with a larger sample size. Another difficulty concerns measuring family relation itself. Self-report studies may be biased and give less objective information. However, subjective perception is critical for the development. Furthermore, longitudinal studies are necessary to investigate whether the attachment abnormalities precede the disorder. Since the questionnaires were filled in at home, it could not be ruled out that parents were present when children were completing the questionnaire. This could have implications on the study results.

Despite these limitations, we think that the present results are useful for a new comprehension of family relationships within SM. If confirmed, our findings may also have clinical implications [7], especially for the needs the fathers can have. An important focus of research should be directed at understanding family relationships in SM and incorporating this in therapeutic interventions.

## Figures and Tables

**Table 1:** Demographics [31,32].

	Selective Mutism (SM)	Control Group
	(*n* = 28)	(*n* = 33)
	M (SD)	M (SD)
age	12.66 (3.98)	12.45 (3.18)
age of SM onset:	3.24 (1.26)	
age of SM diagnosis:	7.70 (4.28)	
duration of SM:	9.04 (4.44)	
sex (female/male)	(18/10)	(21/12)
mother tongue: German	25	32
Developmental specifics during infancy and toddler age		
emotional problems	4	0
sleeping problems	3	1
motor developmental delay	5	0
speech developmental delay	3	0
Comorbid diagnoses		
anxiety	4	0
depression	4	0
Read and Spelling Disorder	1	0
ADS/ADHS	1	2

**Table 2:** Family relationship structure—mother’s perspective; compared between the selective mutism group (MG) and the control group (CG).

Samples	MG	CG
	*n* = 28	*n* = 33
**Relationship, mother and child**		
good	92.9% (*n* = 26)	97% (*n* = 32)
mediocre	7.1% (*n* = 2)	3% (*n* = 1)
bad	0% (*n* = 0)	0% (*n* = 0)
**Relationship, father and child**		
good	45% (*n* = 13)	75.8% (*n* = 25)
mediocre	25% (*n* = 7)	6.1% (*n* = 2)
bad	28.6% (*n* = 8)	18.2% (*n* = 6)

**Table 3:** Results of the “Parental-Representation-Screening-Questionnaire” for the group with selective mutism (MG) compared with the control group (CG).

Samples	Mother/Father	MG	CG	
	M/F	M (SD)	M (SD)	*p* Values
Scores				
cohesion	M	2.97 (0.68)	3.11 (0.73)	
	F	2.05 (1.07)	2.78 (1.01)	F (1, 34) = 6.43, *p* < 0.05, d = 0.76
identification	M	2.39 (0.63)	2.69 (0.76)	
	F	1.95 (0.91)	2.72 (0.84)	X^2^ (1) = 6.23, *p* < 0.01, d = 0.88
autonomy	M	2.42 (0.70)	2.68 (0.65)	
	F	2.13 (0.76)	2.64 (0.74)	X^2^ (1) = 11.01, *p* = 0.001, d = 0.67
conflict	M	1.32 (0.77)	1.56 (0.71)	
	F	1.38 (0.91)	1.21 (0.66)	
punishment	M	0.31 (0.53)	0.19 (0.41)	
	F	0.11 (0.22)	0.17 (0.44)	
rejection	M	0.19 (0.43)	0.18 (3.77)	
	F	0.42 (0.84)	0.18 (0.36)	
emot. appropr.	M	0.80 (0.51)	0.76 (0.63)	
	F	0.22 (0.35)	0.34 (0.43)	
overprotection	M	1.92 (0.87)	1.66 (0.90)	
	F	1.20 (0.81)	1.30 (0.94)	
need for help	M	1.34 (0.86)	1.23 (0.69)	
	F	0.85 (0.64)	0.69 (0.53)	
index value	M	3.26 (2.62)	4.11 (3.02)	
	F	2.81 (3.08)	4.94 (3.28)	X^2^ (1) = 4.71, *p* < 0.05, d = 0.67

M = Mother; F = Father.

## Data Availability

Not applicable.

## References

[1] American Psychiatric Association (2013). Diagnostic and Statistical Manual of Mental Disorders (DSM-5).

[2] Hartmann B. (2019). Gesichter des Schweigens—Systemische Mutismus-Therapie/SYMUT als Therapiealternative.

[3] Muris P., Ollendick T. (2015). Children who are anxious in silence: A review on selective mutism, the new anxiety disorder in DSM-5. Clin. Child Fam. Psychol. Rev..

[4] Bergman R.L., Piacentini J., McCracken J.T. (2002). Prevalence and description of selective mutism in a school-based sample. J. Am. Acad. Child Adolesc. Psychiatry.

[5] Koop S., Gillberg C. (1997). Selective mutism: A population-based study: A research note. J. Child Psychol. Psychiatry.

[6] Kumpulainen K., Räsänen E., Raaska H., Somppi V. (1998). Selective mutism among second-graders in elementary school. Eur. Child Adolesc. Psychiatry.

[7] Wichtmann A. (2011). Mutismus im System—System im Mutismus? Logopädisch-systemische Betrachtungen des kindlichen Selektiven Mutismus. Forum Logop..

[8] Capozzi F., Manti F., Di Trani M., Romoni M., Vigilante M., Sogos C. (2017). Children’s and parent’s psychological profiles in selective mutism and generalized anxiety disorder: A clinical study. Eur. Child Adolesc. Psychiatry.

[9] Stein M.B., Yang B.Z., Chavira D.A., Hitchcock C.A., Sung S.C., Shipon-Blum E., Gelernter J.A. (2011). Common Genetic Variant in the Neurexin Superfamily Member CNTNAP2 Is Associated with Increased Risk for Selective Mutism and Social Anxiety-Related Traits. Biol. Psychiatry.

[10] Ambrosino S., Alessi M. (1979). Elective mutism: Fixation and the double bind. Am. J. Psychoanal..

[11] Perednik R. (2012). An Interview with Ruth Perednik: Treating Selective Mutism. http://www.freepatentsonline.com/article/North-American-Journal-Ps.

[12] Steinhausen H.C., Adamek R. (1997). The family history of children with elective mutism: A research report. Eur. Child Adolesc. Psychiatry.

[13] Chavira D., Shipon-Blum E., Hitchcock C., Cohan S., Stein M.B. (2007). Selective Mutism and Social Anxiety Disorder: All in the Family?. J. Am. Acad. Child Adolesc. Psychiatry.

[14] Alyanak B., Kılıncaslana A., Harmancıa H.S., Demirkayab S.K., Yurtbaya T., Vehid H.E. (2013). Parental adjustment, parenting attitudes and emotional and behavioral problems in children with selective mutism. J. Anxiety Disord..

[15] Koskela M., Roshan C., Luntamo T., Suominen A., Steinhausen H.C., Sourander A. (2020). The impact of parental psychopathology and sociodemographic factors in selective mutism—A nationwide population-based study. BMC Psychiatry.

[16] Anstendig K.D. (1999). Is selective mutism an anxiety disorder? Rethinking DSM-IV classification. J. Anxiety Disord..

[17] Brown J.B., Lloyd H. (1975). A controlled study of children not speaking at school. J. Assoc. Work. Maladjust. Child..

[18] Edison S.C., Evans M.A., McHolm A.E., Cunningham C.E., Nowakowski M.E., Boyle M. (2011). An investigation of control among parents of selectively mute, anxious, and non-anxious children. Child Psychiatry Hum. Dev..

[19] Elizur Y., Perednik R. (2003). Prevalence and description of selective mutism in immigrant and native families: A controlled study. J. Am. Acad. Child Adolesc. Psychiatry.

[20] Goll K. (1979). Role structure and subculture in families of elective mutists. Fam. Process.

[21] Remschmidt H., Poller Herpertz-Dahlmann B., Hennighausen K., Gutenbrunner C. (2001). A follow-up study of 45 patients with selective mutism. Eur. Arch. Psychiatry Clin. Neurosci..

[22] Rolf M.K. (2019). Fathers and mutism—A research of attachment theory. Sprache Stimme Gehör.

[23] Sharkey L., McNicholas F. (2008). ‘More than 100 years of silence’, elective mutism: A review of the literature. Eur. Child Adolesc. Psychiatry.

[24] Wilkins R. (1985). A comparison of elective mutism and emotional disorders in children. Br. J. Psychiatry.

[25] Wright H.L. (1968). A clinical study of children who refuse to talk in school. J. Am. Acad. Child Psychiatry.

[26] Buzzella B., Ehrenreich-May J., Pincus D.B. (2011). Comorbidity and Family Factors Associated with Selective Mutism. Child Dev. Res..

[27] Compare A., Brugnera A., Zarbo C., Partucelli M. (2017). Structural analyses of members’ relationships in a selective mutism family: A single case study. Adv. Psychol. Res..

[28] Vasilyeva N. (2013). Significant factors in the development of elective mutism: A single case study of a 5 year-old girl. Br. J. Psychother..

[29] Cunningham C.E., McHolm A., Boyle M.H., Patel S. (2004). Behavioral and emotional adjustment, family functioning, academic performance, and social relationships in children with selective mutism. J. Child Psychol. Psychiatry.

[30] Yeganeh R., Beidel D.C., Turner S. (2006). Selective mutism: More than social anxiety?. Depress. Anxiety.

[31] Melfsen S., Jans T., Romanos M., Walitza S. (2022). Emotion regulation in selective mutism: A comparison group study in children and adolescents with selective mutism. J. Psychiatr. Res..

[32] Melfsen S., Romanos M., Jans T., Walitza S. (2021). Betrayed by the nervous system: A comparison group study to investigate the ‘unsafe world’ model of selective mutism. J. Neural. Transm..

[33] Bergman R.L., Keller M.L., Piacentini J., Bergman A.J. (2008). The Development and Psychometric Properties of the Selective Mutism Questionnaire. J. Clin. Child Adolesc. Psychol..

[34] Letamendi A.M., Chavira D.A., Hitchcock C.A., Roesch S.C., Shipon-Blum E., Stein M.B. (2008). Selective Mutism Questionnaire: Measurement Structure and Validity. J. Am. Acad. Child Psychiatry.

[35] Oerbeck B., Overgaard K.R., Bergman R.L., Pripp A.H., Kristensen H. (2020). The Selective Mutism Questionnaire: Data from typically developing children and children with selective mutism. Clin. Child Psychol. Psychiatry.

[36] Titze K., Schenck S., Zulauf Logoz M., Lehmkuhl U. (2013). Assessing the Quality of the Parent-Child Relationship: Validity and Reliability of the Child-Parent Relationship Test (ChiP-C). J. Child Fam. Stud..

[37] R Core Team (2014). R: A Language and Environment for Statistical Computing.

[38] Titze K., Lehmkuhl U. (2010). Parental-Representation-Screening-Questionnaire for Children and Adolescents (PRSQ).

[39] Nowakowski M.E., Tasker S.L., Cunningham C.E., McHolm A.E., Edison S., St Pierre J., Boyle M.H., Schmidt L.A. (2011). Joint Attention in Parent–Child Dyads Involving Children with Selective Mutism: A Comparison Between Anxious and Typically Developing Children. Child Psychiatry Hum. Dev..

[40] Albrigtsen V., Eskeland B., Maehle M. (2015). Ties of silence—Family lived experience of selective mutism in identical twins. Clin. Child Psychol. Psychiatry.

[41] Mattejat F. (1993). Subjektive Familienstrukturen—Untersuchungen zur Wahrnehmung der Familienbeziehungen und zu ihrer Bedeutung für die Psychische Gesundheit von Jugendlichen.

[42] Beesdo K., Pine D.S., Lieb R., Wittchen H.U. (2010). Incidence and risk patterns of anxiety and depressive disorders and categorization of generalized anxiety disorder. Arch. Gen. Psychiatry.

